# Effect of climate change on *Clinopodium polycephalum* (Vaniot) C. Y. Wu & S. J. Hsuan distribution adopting temporal data, ArcGIS, and the MaxEnt model

**DOI:** 10.3389/fpls.2024.1445764

**Published:** 2024-09-09

**Authors:** Zongran Lu, Yuxin Shan, Huijiao Shan, Haicheng Wen, Yanan Wu, Rongchun Han, Xiaohui Tong

**Affiliations:** ^1^ School of Pharmacy, Anhui University of Chinese Medicine, Hefei, China; ^2^ Department of Food and Drug Inspection and Testing, Huludao City Inspection and Testing Center, Huludao, China; ^3^ Faculty of Zhuang Medicine, Guangxi University of Chinese Medicine, Nanning, China; ^4^ Joint Research Center for Chinese Herbal Medicine of Anhui of IHM, Anhui University of Chinese Medicine, Hefei, China; ^5^ School of Life Sciences, Anhui University of Chinese Medicine, Hefei, China

**Keywords:** *Clinopodium polycephalum*, biodiversity bioinformatics, environment variables, climate change, habitat distribution

## Abstract

*Clinopodium polycephalum* (Vaniot) C. Y. Wu & S. J. Hsuan, a vital plant in traditional Chinese medicine, has been used for its hemostatic properties since 1220 AD. Despite its recognized medicinal benefits including anti-inflammatory and cardiovascular applications and increasing market demands, research on this plant remains limited, particularly from the perspective of plant ecology. Due to global warming and the resultant climate change, studies on the distribution and conservation of *C. polycephalum* are of great importance, especially when a clear trend that its habitat shifts to the north was observed. To predict the potential distribution of *C. polycephalum* under distinct climate situations, the MaxEnt model was used along with the ArcGIS software. As a result, an AUC value of 0.931 was achieved, indicating high predictive accuracy of the model. By analyzing 135 occurrence points and their corresponding bioclimatic factors (including precipitation), soil data, and other environmental variables (49 in total), 16 key factors including pH value and basic saturation were selected for downstream analysis. It was found that solar radiation in May, precipitation in May and April, and the lowest temperature in the coldest month are important factors influencing the growth and distribution of *C. polycephalum*. Compared to the current climate scenario, the future suitable habitat for *C. polycephalum* is expected to shift northwest, and under the SSP245-2061-2080 climate scenario, its highly suitable habitat area is projected to increase by 886,000 km^2^. These findings provide crucial insights into the environmental drivers of *C. polycephalum* distribution and aid in its preservation and sustainable use in traditional medicine. Based on the findings of this study, future research should focus on factors such as solar radiation in May and the lowest temperature in the coldest month within the suitable habitat to ensure its effective conservation.

## 1 Introduction

*Clinopodium polycephalum* (Vaniot) C. Y. Wu & S. J. Hsuan is a perennial herb from the family Lamiaceae. The plant, locally called “duan xue liu,” has been used in traditional Chinese medicine as a hemostatic agent for more than 800 years (Li et al., 2017). According to Plants of the World Online (https://powo.science.kew.org/), central and south China is the native range of *C. polycephalum* that grows primarily in the temperate biome where the dominant soil types are yellow-brown earth, yellow-cinnamon soil, red earth-yellow earth, and subalpine meadow soil (Liu et al., 2020). It thrives in warm and humid conditions, often found along forest margins and in mountainous or hilly grasslands (Yang et al., 2014), suggesting that temperature, precipitation, solar radiation, and other relevant environmental factors could significantly affect its growth and distribution. Chemical analyses also revealed the presence of saponins, flavonoids, phenylpropanoids, and volatile oils (Wang et al., 2024) within this botanical remedy, underscoring its profound medicinal and economic significance. Despite its potential, studies on “duan xue liu” remain relatively shallow, leaving much of *C. polycephalum*’s value untapped. Moreover, the looming threat of environmental degradation poses a significant risk to its natural growth.

The growth environment profoundly shapes the medicinal potency of traditional Chinese medicinal plants, constituting a pivotal determinant of their efficacy (Kwon et al., 2014; Miao et al., 2019; Yoon et al., 2022; Tian et al., 2023). Environmental variables exert a significant influence over plant growth and development, dictating their geographical spread and population dynamics (Ni and Vellend, 2023; Huang et al., 2024). Greenhouse gas emissions lead to climate change, causing extreme weather events and impacting the growth of plants (Parmesan and Hanley, 2015; Franklin et al., 2016). For instance, global warming reduced the pollen intensity of *Artemisia* in Poland (Piotrowska-Weryszko et al., 2024), and extremely high temperatures could influence the development of *Eriophyton wallichii* extrafloral structures (Peng et al., 2016). Climate change’s effect on *C. polycephalum* is also evident. **The Illustrated Investigation of Plant Names and Facts** recorded the distribution of *C. polycephalum* in Sichuan, Yunnan, and Guizhou provinces (Wu, 1848). However, the *Flora of China* updated its habitat in Sichuan, Yunnan, Guizhou, Henan, Hubei, and Gansu provinces, exhibiting a noticeable northward shift in its distribution (Wu and Li, 1977). So far, there has been no systematic study on the influence of climatic environmental factors on the distribution of *C. polycephalum*. It is therefore necessary to identify the main environmental drivers affecting the species and predict its future suitable habitat to ensure its conservation.

In this study, in addition to climate variables, factors such as soil, solar radiation, and precipitation were also considered. Research showed that drought significantly affected the biomass and nutrient content of Lamiaceae plants (García-Caparrós et al., 2019). The intensity of solar radiation influenced the antioxidant activity of Lamiaceae plants (de Medeiros Gomes et al., 2021), and soil factors impacted the heavy metal absorption and photosynthesis efficiency of *Melissa officinalis* L (Adamczyk-Szabela and Wolf, 2022). Based on the principle of correlation, these environmental factors were incorporated into the MaxEnt distribution prediction model in this study, aiming to achieve a more accurate prediction.

MaxEnt, a versatile and potent probabilistic model, operates on the principle of maximum entropy, leveraging machine learning algorithms to forecast the potential suitability area of a species (Khan et al., 2022). Unlike models constrained by specific probability distribution assumptions such as generalized linear models (GLMs) and generalized additive models (GAMs), MaxEnt is characterized by its flexibility, offering robust analyses by scrutinizing the relationship between known species’ distribution points and environmental variables (Korbel, 2021). By seeking the probability distribution with utmost uncertainty under specified constraints (Phillips et al., 2006; Harte and Newman, 2014), MaxEnt excels in handling biased data, such as limited species occurrence records, and produces visually intuitive probability maps of species existence. Complementing MaxEnt’s prowess, ArcGIS emerges as a formidable GIS platform renowned for its comprehensive spatial analysis capabilities (Oliveira et al., 2020; Fleming et al., 2022). By seamlessly integrating diverse spatial data encompassing climate, elevation, and soil, ArcGIS empowers researchers to forecast species’ potential distribution areas with precision (Cong et al., 2023). Leveraging its advanced spatial analysis and modeling features, ArcGIS facilitates the synthesis of environmental factors to elucidate species habitat preferences and distribution patterns (Rahmanian et al., 2022; Amindin et al., 2024). The synergy between MaxEnt and ArcGIS thus fosters a holistic approach to ecological research, enabling nuanced insights into species ecology and distribution dynamics (Brown et al., 2017).

In the context of this study, the maximum entropy model was employed to forecast the distribution of *C. polycephalum*, offering valuable insights into the environmental factors influencing its resource distribution. This predictive analysis serves as a pivotal reference point for addressing challenges related to *C. polycephalum* habitat alteration and resource distribution. By harnessing the predictive capabilities of the MaxEnt model within the framework of ArcGIS, researchers can proactively strategize conservation initiatives tailored to the unique ecological requirements of *C. polycephalum*, thereby contributing to the sustainable management of traditional Chinese medicinal resources.

## 2 Materials and methods

### 2.1 Geographical distribution data and processing of C*linopodium* polycephalum

**Figure 1:**
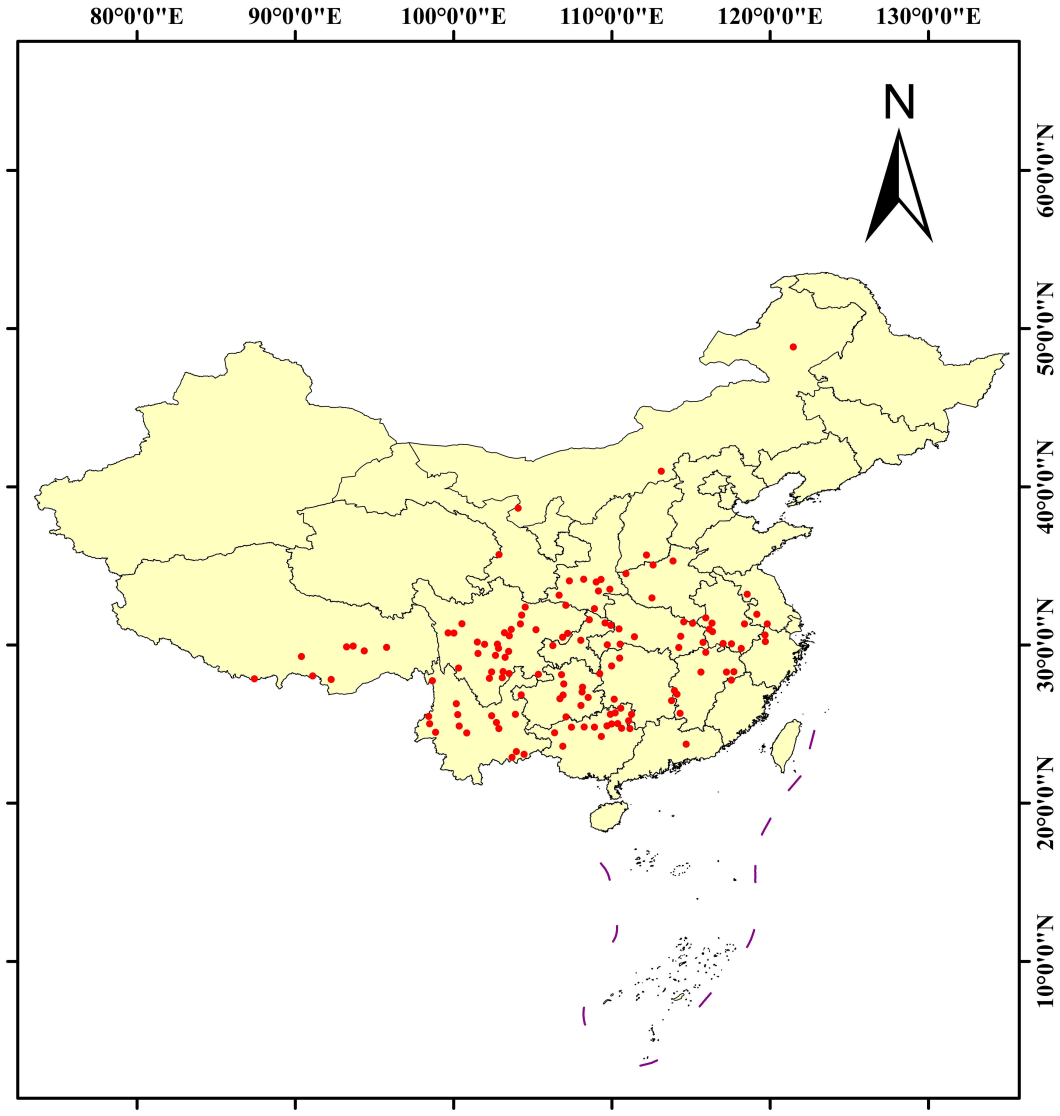
The distribution of *Clinopodium polycephalum* in China, with red dots representing individual distribution coordinate points.

The geographical distribution data of *C. polycephalum* were obtained from the Chinese Virtual Herbarium (https://www.cvh.ac.cn/) and the National Specimen Information Infrastructure (http://www.nsii.org.cn/). Coordinates were retrieved by querying relevant latitude and longitude data through the Baidu Maps (https://map.baidu.com/) website. After removing duplicate coordinates and outdated records (the records of specimens prior to the 21st century), we obtained a total of 172 data points which were subjected to further screening. The accuracy of the data retrieved from the abovementioned databases was confirmed by the authors manually. To mitigate clustering effects during the modeling process, we used the “Spatially Rarefy Occurrence Data for SDMs (Reduce Spatial Autocorrelation)” tool in ArcGIS software to process the collected 172 coordinate files. We set the resolution of the rarefying parameter to 20 km and finally acquired 135 useful coordinates. The distribution results are shown in **Figure 1**.

### 2.2 Environmental variable data and processing

Studies showed that drought, solar radiation, sunshine duration, and other factors had important effects on the distribution and accumulation of active components in Lamiaceae plants (Mansinhos et al., 2024; Pshenichkina, 2022; Lalević et al., 2023). In addition, Wan et al. (2022) selected soil and precipitation factors to predict the distribution of *Clinopodium chinense*. Therefore, this study combined the above factors and added precipitation, soil, and solar radiation into the model. Bioclimatic and precipitation data were chosen for two emission pathways (SSP245 and SSP585) under the present and future BCC-CSM2-MR climate models for the 2050s (2041–2060) and 2070s (2061–2080), respectively. Because climate model predictions are relatively reliable in the medium term (e.g., 2041–2060), they may have greater uncertainty if they extend too far into the future. Moreover, the medium-term forecast can better reflect the potential impact of current policies and actions. The selection of specific time windows such as 2041–2060 and 2061–2080 makes different studies more comparable and coherent (O’Neill et al., 2017). These data, along with solar radiation data and precipitation data, were obtained from the WorldClim website (https://worldclim.org/). Additionally, soil-related data were sourced from the Harmonized World Soil Database (HWSD) website (https://www.fao.org/soils-portal/data-hub/soil-maps-and-databases/harmonized-world-soil-database-v12/en/). A provincial-level administrative map with a scale of 1:4,000,000 was downloaded from the National Basic Geographic Information System of China (http://nfgis.nsdi.gov.cn) for use as an analytical basemap. All the aforementioned data are suitable for establishing the MaxEnt model.

### 2.3 Establishment of the MaxEnt model

The *C. polycephalum* coordinate data and environmental variables were imported into the MaxEnt model. The contribution rates of variables were calculated using the jackknife method. The output format was set to “Logistic,” the output file type was “asc,” and the operation type was “Crossvalidate” (cross-validation method). A random selection of the test set was set to 25%, leaving the rest 75% as the training set. We chose this setting because the test set evaluated the trained model and assessed its generalization ability and it can effectively avoid inflated measures of model performance (Phillips et al., 2017). The training was repeated 10 times. The number of iterations was set to 10,000 to make the model more accurate, while other parameters remained at their default values. This process yielded the contribution values of each environmental factor to the growth of *C. polycephalum*.

There were correlations among the selected 49 natural environmental variables. To avoid overfitting the model due to multicollinearity, collinearity diagnosis of the environmental variables was performed using SPSS 27. When the correlation coefficient between two environmental factors exceeded 0.8, the environmental factor with the lower contribution rate was discarded. Finally, 16 environmental factors were selected. The *C. polycephalum* coordinates and these environmental factors were imported into the MaxEnt software, and the model was run again according to the above settings to obtain the contribution values of each major environmental factor to the distribution of *C. polycephalum*, as shown in **Table 1**.

**Table:** Table 1 The environmental factors and their contribution rates after screening.

Variables	Description	Percent contribution/%
srad5	Solar radiation in May	25.6
prec5	Precipitation in May	16.5
prec4	April precipitation	10.1
bio6	Min temperature of the coldest month	8.1
prec11	November precipitation	7
bio11	Mean temperature of the coldest quarter	6.9
srad7	Solar radiation in July	6
bio7	Temperature annual range (BIO5–BIO6)	4.4
bio9	Mean temperature of the driest quarter	2.9
srad10	Solar radiation in October	2.4
T-BS	Basic saturation	2.1
T-PH-H_2_O	pH	1.9
prec1	January precipitation	1.8
AWC-class	Soil-effective water content	1.8
srad4	Solar radiation in April	1.7
prec3	March precipitation	0.8

## 3 Results

### 3.1 Model prediction results

**Figure 2:**
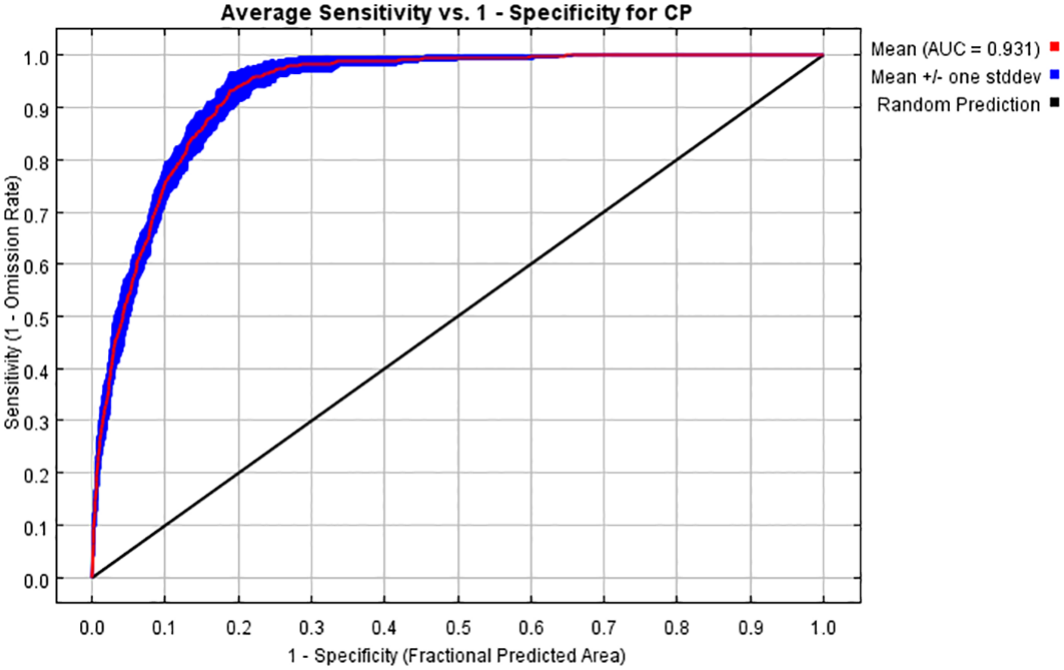
ROC curves of the MaxEnt model for *Clinopodium polycephalum*.

The accuracy of the model predictions can be evaluated using the receiver operating characteristic (ROC) curve and the area under the curve (AUC). The ROC curve is a graphical tool that shows the performance of a classifier at different threshold settings. It helps to understand how models behave in different situations by plotting the relationship between the true-positive rate (TPR) and the false-positive rate (FPR). The AUC is defined as the area under the ROC curve enclosed by the coordinate axes. The value of this area cannot be greater than 1. Since the ROC curve is generally above the line *y* = *x*, the AUC value ranges between 0.5 and 1. The AUC ranges are as follows: 0.5 to 0.6 (fail), 0.6 to 0.7 (poor), 0.7 to 0.8 (fair), 0.8 to 0.9 (good), and 0.9 to 1 (excellent) (Wang et al., 2020). The mean AUC value of the training set in this study was 0.931, as shown in **Figure 2**. In addition, the R program (R Core Team, 2024) was applied to calculate the true skill statistic (TSS) to evaluate the accuracy of MaxEnt’s prediction (Allouche et al., 2006). The evaluation criteria for TSS are categorized into five levels: excellent (0.85–1.0), very good (0.7–0.85), good (0.55–0.7), fair (0.4–0.55), and fail (<0.4). By running R, the TSS value obtained in this study was 0.568. This indicated that the model performed well in predicting results and suggested its suitability for studying the habitat distribution of *C. polycephalum*.

### 3.2 Analysis of dominant environmental variables influencing the distribution of C*linopodium* polycephalum

**Figure 3:**
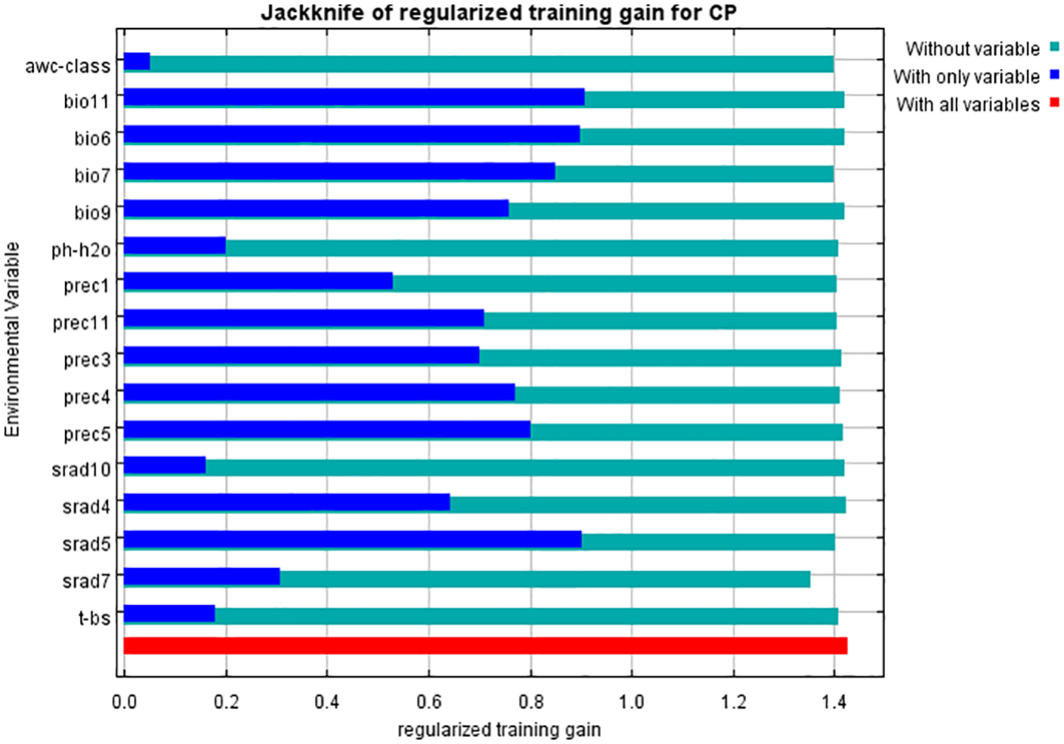
Jackknife test of environmental variables for *Clinopodium polycephalum*. Blue bars indicate only the designated variable, green bars indicate no such variable, and red bars indicate all variables.

The contribution rates of environmental factors were determined using the jackknife method which is a commonly used statistical experiment approach to calculate the overall statistical indicators. It samples a large amount of data from the population, constructs different small samples, and then analyzes and evaluates the population by comparing different dimensions. The conclusions obtained can be very effective in calculating the index of the population. The results of assessing the gain effect of environmental variables through the jackknife method are shown in **Figure 3**. From the graph, it was evident that the mean temperature of the coldest quarter (bio11) and the solar radiation in May (srad5) had the greatest impact on the distribution of *C. polycephalum*. This indicated that these two environmental factors played a decisive role in determining suitable habitats for *C. polycephalum*. Following closely were the minimum temperature of the coldest month (bio6) and the temperature annual range (bio7), which also have significant impacts. Additionally, precipitation in March, April, May, and November, as well as the mean temperature of the driest quarter (bio9), also influenced the distribution of *C. polycephalum*. On the other hand, soil-available water content (AWC-class), soil acidity (pH-H_2_O), basic saturation (T-BS), and solar radiation in July and October contributed relatively less to the distribution of *C. polycephalum*.

**Figure 4:**
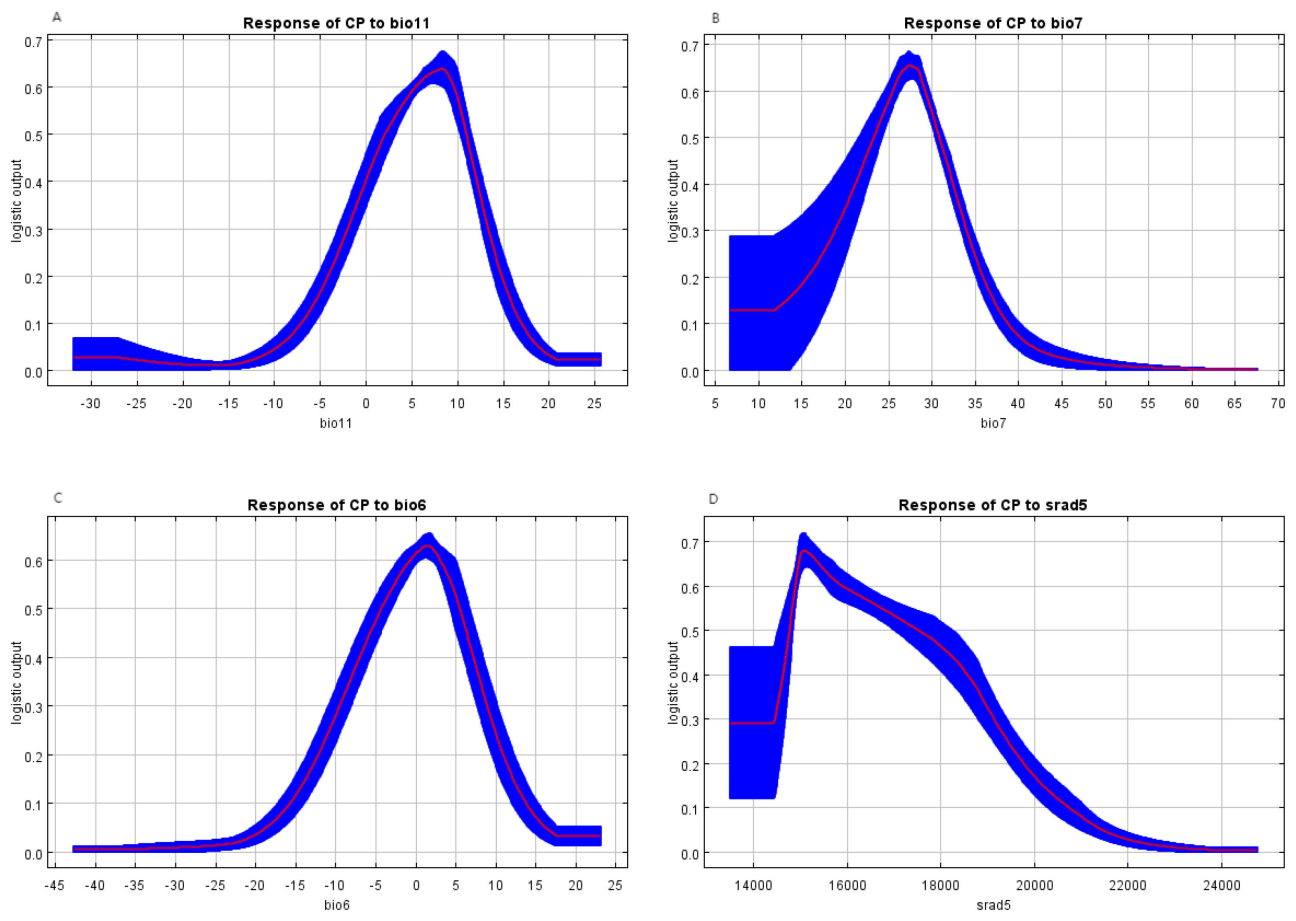
Response curves for the critical environmental variables. **(A)** Mean temperature of the coldest quarter indicates the most suitable temperature of 8°C; **(B)** the annual temperature range suggests that 27°C is the best; **(C)** the minimum temperature of the coldest month demonstrates that the appropriate one is 2°C; **(D)** solar radiation in May proposes that 15,000 kJ m^−2^ day^−1^ is most conducive to the growth of *Clinopodium polycephalum*.

Considering both the modeling contribution rates and impact weights, the relationship between species distribution and environmental factors is reflected through the response curves of the MaxEnt model. The response curves of several environmental factors that significantly influence the distribution of *C. polycephalum* are shown in **Figure 4**. When a factor exceeds 0.5, it can be considered to have an impact on the organism (Xu et al., 2023). In the graph, the mean temperature of the coldest quarter (bio11) is beneficial for the presence of *C. polycephalum* when it falls between 2°C and 11°C. Similarly, the temperature annual range (bio7) between 24°C and 31°C is also favorable for the presence of *C. polycephalum*. Additionally, the minimum temperature of the coldest month should range from −5°C to 5°C, and solar radiation in May between 14,800 and 17,550 kJ m^−2^ day^−1^ is conducive to the growth of *C. polycephalum*. It can be observed that the growth of *C. polycephalum* is mainly associated with solar radiation and temperature.

### 3.3 Potential geographic distribution and habitat evaluation

#### 3.3.1 Current potential distribution of *Clinopodium polycephalum*

**Figure 5:**
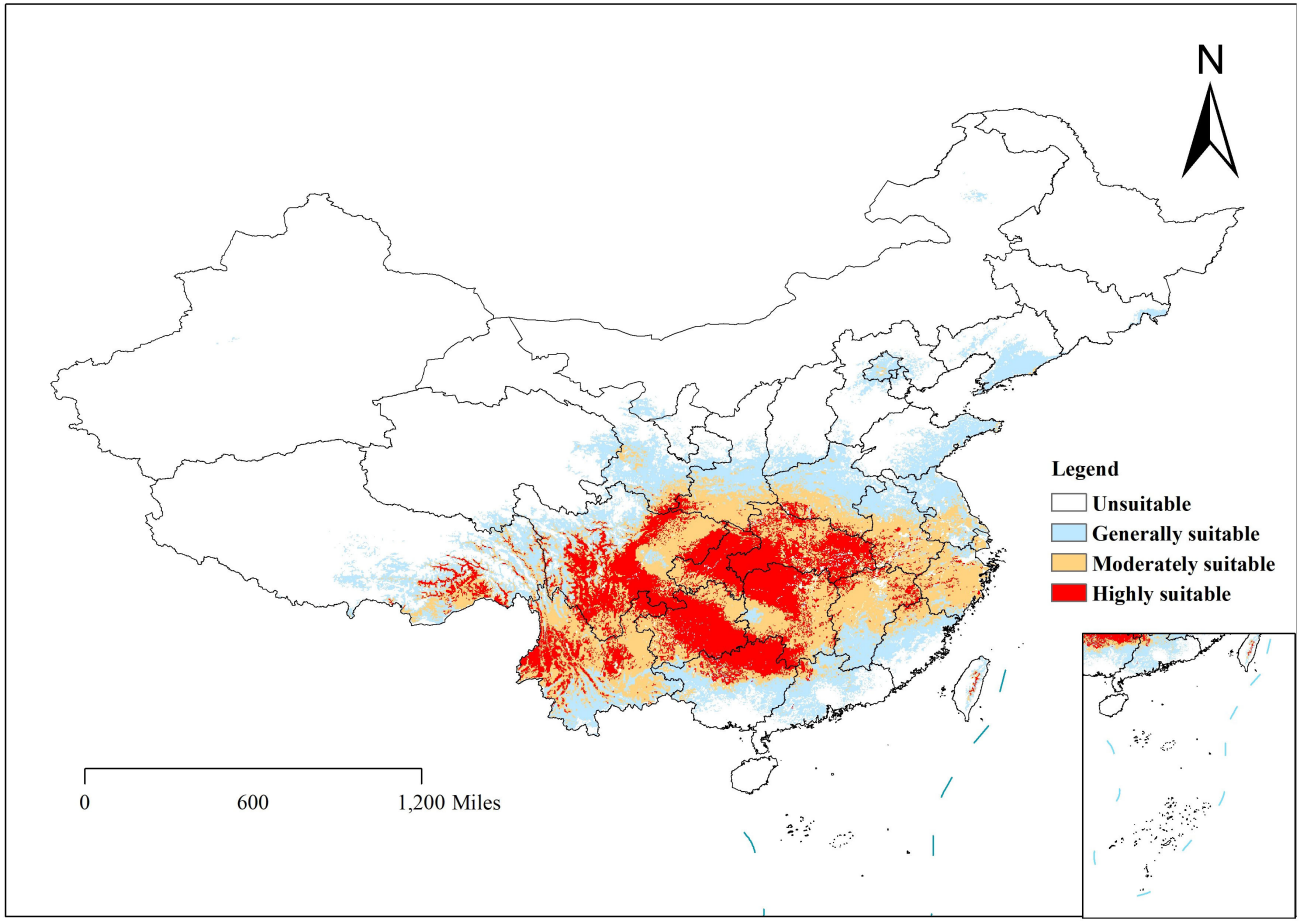
Distribution of *Clinopodium polycephalum* in suitable areas in China. White indicates an unsuitable growth area, and red indicates a highly suitable area.

Depending on the direction of habitat suitability trends, species ranges may shrink or expand. Range shrinkage usually leads to increased vulnerability and potential extinction, while expansion can lead to the colonization of new regions (Parmesan and Yohe, 2003). Shifts in the center of gravity of plant growth can indicate how species respond to climate change. For example, in the Northern Hemisphere, a northward shift in the center of gravity usually indicates that species are migrating to cooler areas in response to rising temperatures (Chen et al., 2011). Understanding the center of gravity displacement can help develop effective conservation strategies. Under the influence of natural environmental factors, the potential distribution of *C. polycephalum* is depicted in **Figure 5**. Using the ArcGIS software, the study determined that *C. polycephalum* had a potential distribution area of approximately 297.6 × 10^4^ km^2^ in the current period, accounting for approximately 31.0% of China’s land area. Among these, the high-suitable habitat zone covered approximately 75.8 × 10^4^ km^2^, primarily concentrated in southern China. It was distributed throughout the entire province of Hubei, the central-northern part of Chongqing Municipality, the northwest part of Hunan Province, the northern part of Guangxi Province, the central part of Guizhou Province, the southeastern part of Sichuan Province, the eastern part of the Tibet Autonomous Region, the northwest corner of Yunnan Province, and areas such as Anhui, Henan, Zhejiang, Jiangxi, and Guangdong. The medium-suitable habitat zone covered approximately 112.3 × 10^4^ km^2^, mainly spreading outward from the high-suitable habitat zone. It was distributed in the central-southern part of Hunan Province, the central-northern part of Jiangxi Province, the central-southern part of Anhui Province, the entire province of Zhejiang, the southern part of Henan Province, and parts of Guizhou, Hubei, Yunnan, Tibet, Gansu, and Sichuan. The low-suitable habitat zone similarly expanded outward from the edge of the medium-suitable habitat zone, covering approximately 109.4 × 10^4^ km^2^. It was primarily distributed in the southern edge of Yunnan, the southern part of Guangxi, the central-northern part of Fujian, Jiangsu, Anhui, the northern part of Henan, the central part of Shaanxi, the southern part of Gansu, and sporadically distributed in provinces such as Shandong, Shanxi, Liaoning, Beijing, and Hebei.

#### 3.3.2 Potential distribution of *Clinopodium polycephalum* under future climate

**Figure 6:**
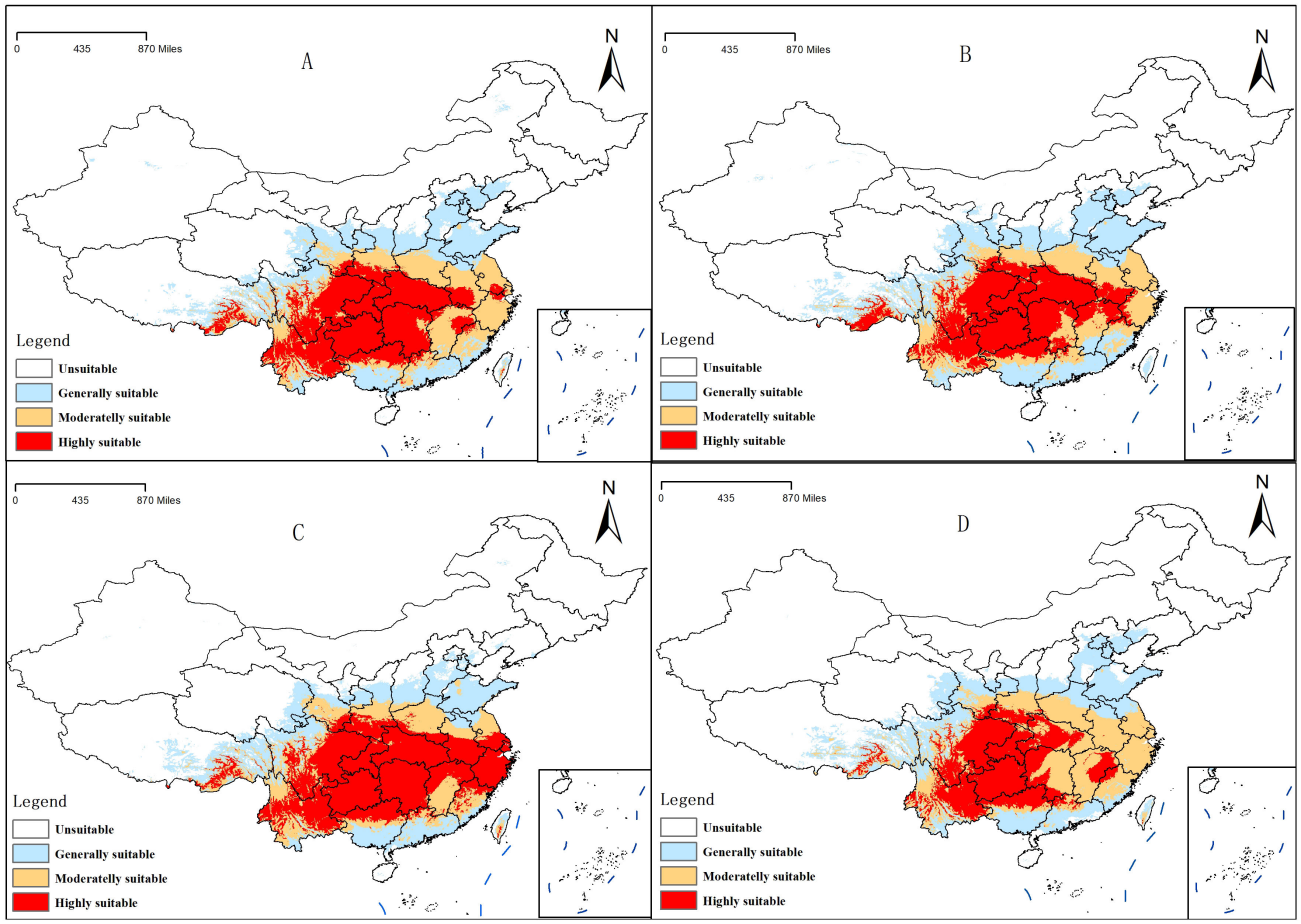
Distribution of suitable areas of *Clinopodium polycephalum* under future climatic conditions. **(A)** 2041–2060, SSP245; **(B)** 2041–2060, SSP585; **(C)** 2061–2080, SSP245; **(D)** 2061–2080, SSP585.

**Figure 7:**
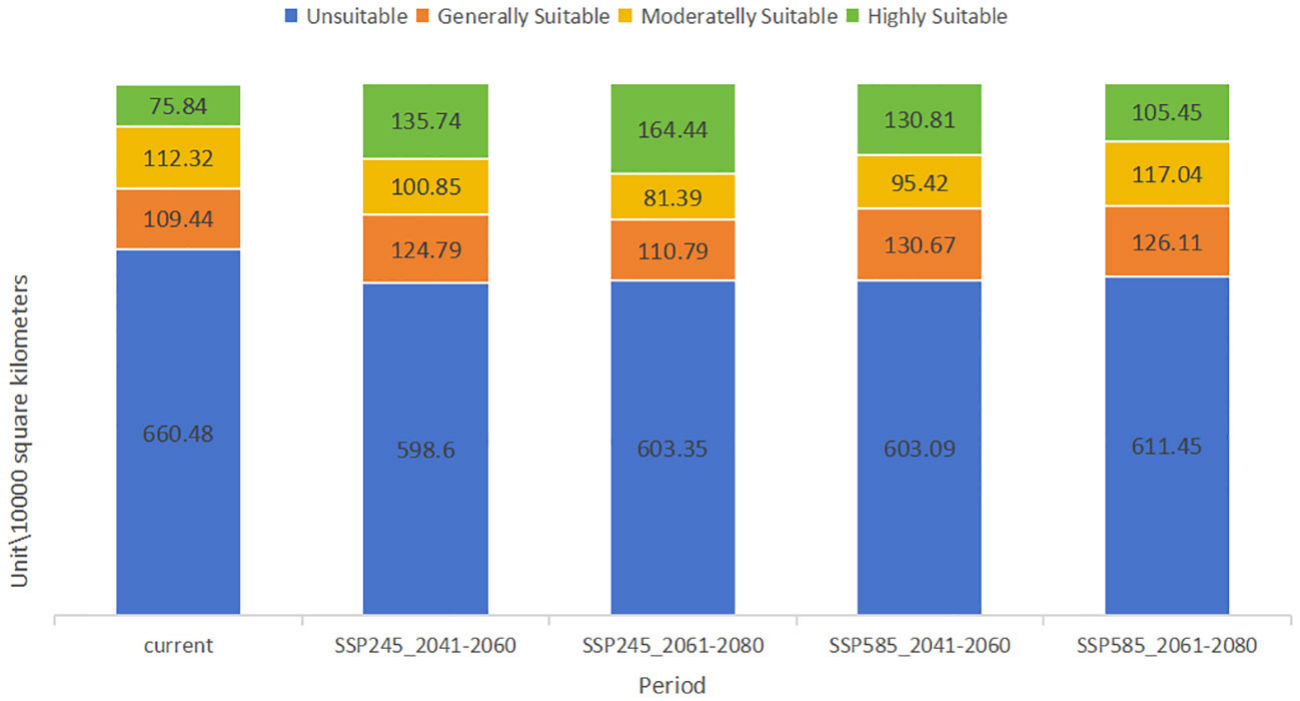
Comparison of the area of each suitable area of *Clinopodium polycephalum* in each period.

The study considered climate conditions under the SSP245 and SSP585 scenarios for the periods 2041–2060 and 2061–2080. After running MaxEnt and ArcGIS, the distribution maps of future *C. polycephalum* were obtained, as shown in **Figure 6**. Compared to the current climate conditions, the high-suitable habitat areas for *C. polycephalum* increase under both future scenarios. Under the SSP245 scenario for the period of 2041–2060 (**Figure 6A**), the high-suitable habitat area for *C. polycephalum* is approximately 135.7 × 10^4^ km^2^, which is 60.0 × 10^4^ km^2^ larger than the current high-suitable habitat area. It is mainly distributed in the southeastern part of Sichuan, Hubei, Chongqing, Guizhou, the northwestern part of Hunan, southern Anhui, and northeastern Yunnan. The middle suitable living area is approximately 100.8 × 10^4^ km^2^, and the low-suitable habitat area is approximately 124.7 × 10^4^ km^2^. The suitable habitat distribution under the SSP585 scenario for the period of 2041–2060 (**Figure 6B**) is similar to that under the SSP245 scenario for the same period. The areas of low-, medium-, and high-suitable habitats are approximately 130.6 × 10^4^, 95.4 × 10^4^, and 130.0 × 10^4^ km^2^, respectively, with generally similar provincial distributions. Under both SSP245 and SSP585 scenarios, the suitable habitat area of *C. polycephalum* for the period of 2061–2080 significantly increases compared to the current conditions. Under the SSP245 scenario, the total suitable habitat area for the period of 2061–2080 is approximately 356.6 × 10^4^ km^2^, with a high-suitable habitat area of approximately 164.4 × 10^4^ km^2^. It is mainly distributed in Zhejiang, southern Anhui, Hubei, Hunan, Guizhou, Chongqing, and southeastern Sichuan. This area not only shows a significant increase compared to the current suitable habitat area but also exhibits a substantial increase compared to the period of 2041–2060 under the SSP245 scenario. However, the areas of low- and medium-suitable habitats have significantly decreased. Under the SSP585 scenario, the situation is slightly different. The high-suitable habitat area for the period of 2061–2080 is approximately 105.3 × 10^4^ km^2^, which is an increase of approximately 30.0 × 10^4^ km^2^ compared to the current climate. However, compared to the period of 2041–2060, the high-suitable habitat area has decreased by approximately 25.0 × 10^4^ km^2^. The comparison of suitable areas in different periods is shown in **Figure 7**.

#### 3.3.3 Changes in the distribution centroid of suitable habitat zones

**Figure 8:**
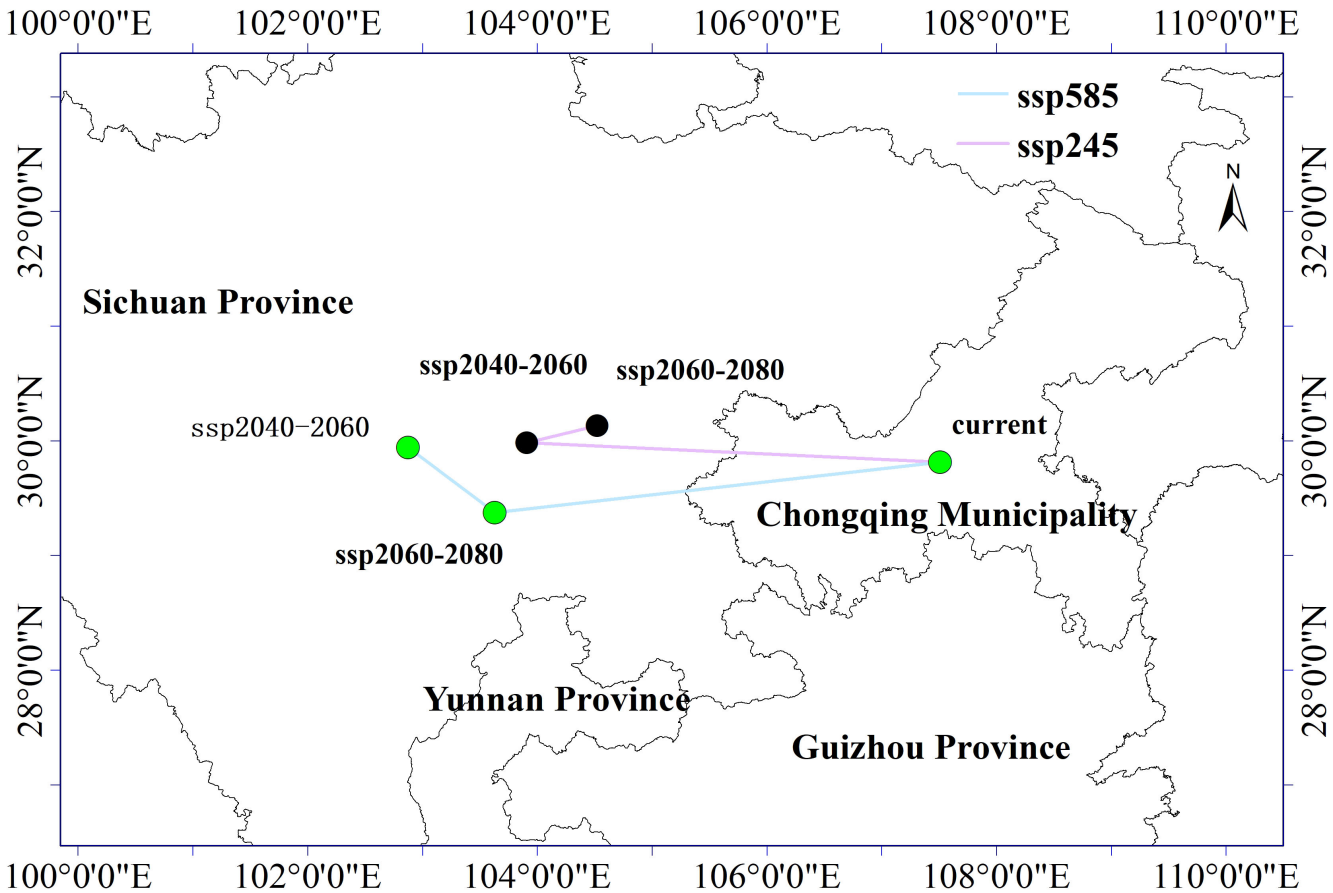
Shift map of the center of gravity in the suitable area of *Clinopodium polycephalum* under future climate scenario.

The prediction results have shown certain differences in the migration of suitable habitat zones for *C. polycephalum* under different climate scenarios (**Figure 8**). Under current climate conditions, the centroid of the suitable habitat zone for *C. polycephalum* growth was located near Fuling District, Chongqing Municipality (107.51°E, 29.81°N). Under the influence of climate change, the centroid of the suitable growth zone for *C. polycephalum* in the period of 2041–2060 under the SSP245 climate scenario moved westward to Meishan City, Sichuan Province (112.8°E, 33.60°N), with a migration distance of 347.3561 km. Similarly, under the same climate scenario for the period of 2061–2080, the centroid of the suitable habitat zone for *C. polycephalum* shifted toward the northeast direction, with a smaller migration distance of 61.4186 km.

Under the SSP585 climate scenario, during the period of 2041–2060, the centroid of the suitable growth zone for *C. polycephalum* generally moved westward to Tianquan County, Ya’an City, Sichuan Province, with a migration distance of 447.1534 km. In the period of 2061–2080, the suitable growth zone for *C. polycephalum* showed slight differences compared to the SSP245 climate scenario, with its centroid mainly shifted toward the northwest direction to Leshan City, Sichuan Province, with a migration distance of 88.0001 km. It can be observed that under future climate change scenarios, the centroid of the suitable habitat zone for *C. polycephalum* tended to migrate westward.

## 4 Discussion

With the exacerbation of global warming, climate conditions are gradually deteriorating, making it crucial to anticipate the distribution trends of species under future climate change, especially for the conservation of biodiversity (Huang, 2011; Du et al., 2022; Kusumoto et al., 2023). Such predictions can assist us in better understanding the adaptability and vulnerability of different species, thereby enabling us to implement appropriate conservation measures. In China, “duan xue liu” has a history of over a thousand years and still holds significant medicinal value today. It is essential to make protective distribution predictions for its parent plant, *C. polycephalum*. This study used the MaxEnt and ArcGIS models to simulate the potential distribution of *C. polycephalum* within China, revealing the impact of climate change on its future growth distribution and identifying its most suitable habitats and priority conservation areas. These findings will contribute to the conservation of *C. polycephalum* resources and help regulate the ecological balance (Zou et al., 2023; Eyre et al., 2024; Zou et al., 2024).

The operational results of this study indicated that under current climate conditions, the primary environmental factors influencing the distribution of *C. polycephalum* were temperature and solar radiation, followed by precipitation, while soil factors had the least impact on *C. polycephalum* distribution. Specifically, solar radiation in May has the highest contribution rate to the distribution of *C. polycephalum*, accounting for 25.6% of the total among the 16 environmental factors selected. The combined contribution rates of solar radiation in May and precipitation in May and April account for 52.2% of the total. The jackknife analysis showed that among the four environmental factors that had a significant impact on *C. polycephalum*, three were related to temperature, suggesting that temperature may play a decisive role in *C. polycephalum* growth. Although direct evidence for *C. polycephalum* is currently unavailable from the literature, studies showed that the optimal germination temperature for the seeds of Lamiaceae plant *Clinopodium sandalioticum* was approximately 20°C–25°C, and cold stratification was detrimental to seed germination at 10°C (Mattana et al., 2016). For the Lamiaceae plant *Origanum compactum*, the optimal germination temperature was 15°C (Laghmouchi et al., 2017). Based on this, it could be inferred that temperature conditions might also have a significant impact on the germination of *C. polycephalum*, which would greatly affect the distribution of *C. polycephalum*. Combining the response curves of these four environmental factors, we concluded that the minimum temperature for *C. polycephalum* growth should not drop below −5°C, the average temperature of the coldest quarter should be between 2°C and 11°C, and the annual temperature difference was preferably between 24°C and 31°C. These conditions may be conducive to *C. polycephalum* growth. The specific impact of solar radiation on *C. polycephalum* is currently unclear. However, studies showed that solar radiation was a key environmental signal regulating plant secondary metabolism. The interaction between ultraviolet-B and photosynthetically active radiation could affect the yield of monoterpenes in mint leaves (Behn et al., 2010), and this may adversely affect its growth. Therefore, it is reasonable that solar radiation also plays a significant role for the growth and distribution of *C. polycephalum*, which is in line with our findings that solar radiation in May was determined as a key factor. Moreover, solar radiation levels between 14,800 and 17,550 kJ m^−2^ day^−1^ may suit the growth requirements of *C. polycephalum*. These conditions align with *C. polycephalum*’s preference for light, its strong adaptability and tolerance to the environment, and its relatively low soil requirements (Ye et al., 2022). Precipitation may be related to the synthesis of phenolic compounds in *Hyptis* Jacq. (Lamiaceae), and the population from the Atlantic forest contains higher phenolic compounds than that from the Brazilian Serrado (dos Santos et al., 2018). Rainfall in different seasons and locations may well have an effect on *C. polycephalum*. Our model forecasted that precipitation in April and May should be no less than 60 and 90 mm, respectively. External precipitation requirements we see could be the result of the internal physiological needs of *C. polycephalum*, which merits future studies.

Currently, the potential suitable habitat for *C. polycephalum* was concentrated in the southern regions of China, primarily in Sichuan Province, Chongqing Municipality, Anhui Province, Guizhou Province, and Zhejiang Province, among others. Secondary distributions were observed in provinces such as Henan, Gansu, Guangdong, and Guangxi Zhuang Autonomous Region. This distribution pattern was largely consistent with actual distribution areas (Huang et al., 1991), indicating a certain degree of accuracy in the model. Future habitat suitability models predicted that under the SSP245 scenario, the overall area of suitable habitat for *C. polycephalum* will increase in future periods, with an increase in high-suitable habitat areas. This suggested that *C. polycephalum* in China had not yet reached its maximum distribution range and will continue to expand under the backdrop of global climate warming. However, under the SSP585 scenario, the area of suitable habitat for *C. polycephalum* in 2061–2080 decreased slightly compared with that in 2041–2060, with a significant reduction in the high-suitable habitat area. This may indicate that under this scenario, climate change may not favor the growth of *C. polycephalum*.

In the process of adapting to climate change, many species tend to migrate toward higher latitudes (Nuñez et al., 2023). This study analyzed the centroid of the suitable habitat zone for *C. polycephalum* using the ArcGIS software. It was found that under the SSP245 scenario, the centroid of the suitable habitat zone for *C. polycephalum* generally shifted toward higher latitudes with a “westward” trend. Under the SSP585 scenario, from 2041 to 2060, the centroid shifted slightly toward higher latitudes, while from 2061 to 2080, there was a slight shift toward lower latitudes, with an overall westward trend. However, from 2061 to 2080, the centroid slightly shifted eastward compared to the period from 2041 to 2060.

Although the environmental indicators included in the modeling process encompass climate factors, precipitation, solar radiation, soil, and other factors, there are still certain limitations. Additionally, the choice of research scale is one of the uncertainties in species distribution prediction. Furthermore, climate warming may increase the frequency of extreme weather events, leading to habitat degradation and loss (Callaghan et al., 2004; Zhou et al., 2013). Therefore, the actual distribution of suitable habitat for *C. polycephalum* may be smaller than the predicted results in this study. Based on the study of the potential geographical distribution pattern of *C. polycephalum* in different periods, the suitability of the lowest temperature in the coldest month, the average temperature in the coldest month, the solar radiation in May, and the precipitation in April and May can be considered more in the future protection and planting of *C. polycephalum*, so as to develop corresponding physiological regulation measures and better protect the wild germplasm resources of *C. polycephalum*.

## 5 Conclusion

The rapid climate change caused by global warming has significantly affected many plant species. For *C. polycephalum*, a traditional Chinese medicinal plant with great value, predicting its future habitat is crucial given its inadequate studies and underdeveloped status. This study explored the potential distribution of *C. polycephalum* in China and assessed the impact of climate change using the MaxEnt model and ArcGIS software. The analysis of geographical distribution data and environmental variables revealed that temperature and solar radiation are the primary factors influencing *C. polycephalum*’s suitable habitat under current climatic conditions, with solar radiation in May also playing a pivotal role. Temperature, particularly the mean temperature of the coldest quarter and the minimum temperature, significantly impacts *C. polycephalum*’s growth by potentially affecting its seed germination. While climate change may expand suitable growth areas in the future, it also projects reductions in certain areas, necessitating tailored conservation and management strategies. Our finding provides essential data for the conservation and sustainable management of *C. polycephalum* resources. By studying *C. polycephalum*’s ecology and distribution dynamics, this paper offers valuable insights for future studies and conservation efforts, underscoring the practical significance of adaptive management approaches to ensure the long-term survival and medicinal availability of *C. polycephalum*.

## Data Availability

The original contributions presented in the study are included in the article/supplementary material. Further inquiries can be directed to the corresponding authors.

## References

[B1] Adamczyk-Szabela D. Wolf W. M. (2022). The impact of soil pH on heavy metals uptake and photosynthesis efficiency in *Melissa officinalis*, *Taraxacum officinalis*, *Ocimum basilicum* . Molecules 27, 4671. doi: 10.3390/molecules27154671 35897849 PMC9331646

[B2] Allouche O. Tsoar A. Kadmon R. (2006). Assessing the accuracy of species distribution models: prevalence, kappa and the true skill statistic (TSS). J. Appl. Ecol. 43, 1223–1232. doi: 10.1111/j.1365-2664.2006.01214.x

[B3] Amindin A. Pourghasemi R. H. Safaeian R. Rahmanian S. Tiefenbacher J. Naimi B. (2024). Predicting current and future habitat suitability of an endemic species using data-fusion approach: responses to climate change. Rangeland Ecol. Manag 94, 149–162. doi: 10.1016/j.rama.2024.03.002

[B4] Behn H. Albert A. Marx F. Noga G. Ulbrich A. (2010). Ultraviolet-B and photosynthetically active radiation interactively affect yield and pattern of monoterpenes in leaves of peppermint (Mentha x piperita L.). J. Agric. Food Chem. 58, 7361–7367. doi: 10.1021/jf9046072 20481601

[B5] Brown J. L. Bennett J. R. French C. M. (2017). SDMtoolbox 2.0: the next generation Python-based GIS toolkit for landscape genetic, biogeographic and species distribution model analyses. PeerJ 5, e4095. doi: 10.7717/peerj.4095 29230356 PMC5721907

[B6] Callaghan T. V. Björn L. O. Chernov Y. Chapin T. Christensen T. R. Huntley B.. (2004). Biodiversity, distributions and adaptations of Arctic species in the context of environmental change. Ambio 33, 404–417. doi: 10.1579/0044-7447-33.7.404 15573569

[B7] Chen I. C. Hill J. K. Ohlemüller R. Roy D. B. Thomas C. D. (2011). Rapid range shifts of species associated with high levels of climate warming. Science 333, 1024–1026. doi: 10.1126/science.1206432 21852500

[B8] Cong P. Zhang D. Yi M. (2023). Application of ArcGIS 3D modeling technology in the study of land use policy decision making in China. Sci. Rep. 13, 20695. doi: 10.1038/s41598-023-47171-z 38001099 PMC10674007

[B9] de Medeiros Gomes J. Cahino Terto M. V. Golzio do Santos S. Sobral da Silva M. Fechine Tavares J. (2021). Seasonal variations of polyphenols content, sun protection factor and antioxidant activity of two Lamiaceae species. Pharmaceutics 13, 110. doi: 10.3390/pharmaceutics13010110 33467160 PMC7829895

[B10] dos Santos K. P. Sedano-Partida M. D. Sala-Carvalho W. R. Loureiro B. O. S. J. da Silva-Luz C. L. Furlan C. M. (2018). Biological activity of *Hyptis* Jacq. (Lamiaceae) is determined by the environment. Ind. Crop Prod 112, 705–715. doi: 10.1016/j.indcrop.2017.12.065

[B11] Du Y. J. Zhao Y. Dong S. P. Chen G. K. Wang X. Y. Ma K. P. (2022). The diversity distribution and climatic niche of Samara species in China. Front. Plant Sci. 13. doi: 10.3389/fpls.2022.895720 PMC9249021 35783943

[B12] Eyre D. André E. M. Castro K. Dale C. Fowler G. Hess B.. (2024). International collaboration to assess and manage the impacts of climate change on plant health in the framework of the International Plant Protection Convention. Bull. OEPP 54, 89–91. doi: 10.1111/epp.12987

[B13] Fleming J. Marvel S. W. Supak S. Motsinger-Reif A. A. Reif D. M. (2022). ToxPi*GIS Toolkit: creating, viewing, and sharing integrative visualizations for geospatial data using ArcGIS. J. Expo Sci. Environ. Epidemiol. 32, 900–907. doi: 10.1101/2021.10.08.21264756 35474345 PMC9039976

[B14] Franklin J. Serra-Diaz J. M. Syphard A. D. Regan H. M. (2016). Global change and terrestrial plant community dynamics. Proc. Natl. Acad. Sci. U.S.A. 113, 3725–3734. doi: 10.1073/pnas.1519911113 26929338 PMC4833242

[B15] García-Caparrós P. Romero M. J. Llanderal A. Cermeño P. Lao M. T. Segura M. L. (2019). Effects of drought stress on biomass, essential oil content, nutritional parameters, and costs of production in six Lamiaceae species. Water 11, 573. doi: 10.3390/w11030573

[B16] Harte J. Newman E. A. (2014). Maximum information entropy: a foundation for ecological theory. Trends Ecol. Evol. 29, 384–389. doi: 10.1016/j.tree.2014.04.009 24863182

[B17] Huang H. W. (2011). Plant diversity and conservation in China: planning a strategic bioresource for a sustainable future. Bot. J. Linn Soc. 166, 282–300. doi: 10.1111/j.1095-8339.2011.01157.x 22059249

[B18] Huang E. H. Chen Y. X. Yu S. X. (2024). Climate factors drive plant distributions at higher taxonomic scales and larger spatial scales. Front. Ecol. Evol. 11. doi: 10.3389/fevo.2023.1233936

[B19] Huang S. H. Hu J. L. Jing C. Q. (1991). Research report on the original plant of “Duan xue liu. J. Anhui Univ Chin. Med. 4, 57–60.

[B20] Khan A. M. Li Q. Saqib Z. Khan N. Habib T. Khalid N.. (2022). MaxEnt modelling and impact of climate change on habitat suitability variations of economically important Chilgoza Pine (*Pinus gerardiana* Wall.) in south Asia. Forests 13, 715. doi: 10.3390/f13050715

[B21] Korbel J. (2021). Calibration invariance of the MaxEnt distribution in the maximum entropy principle. Entropy 23, 96. doi: 10.3390/e23010096 33440777 PMC7826740

[B22] Kusumoto B. Chao A. Eiserhardt W. L. Svenning J. C. Shiono T. Kubota Y. (2023). Occurrence-based diversity estimation reveals macroecological and conservation knowledge gaps for global woody plants. Sci. Adv. 9, eadh9719. doi: 10.1126/sciadv.adh9719 37801494 PMC10558125

[B23] Kwon Y. K. Bong Y. S. Lee K. S. Hwang G. S. (2014). An integrated analysis for determining the geographical origin of medicinal herbs using ICP-AES/ICP-MS and 1 H NMR analysis. Food Chem. 161, 168–175. doi: 10.1016/j.foodchem.2014.03.124 24837936

[B24] Laghmouchi Y. Belmehdi O. Bouyahya A. Senhaji N. S. Abrini J. (2017). Effect of temperature, salt stress and pH on seed germination of medicinal plant *Origanum compactum* . Biocatalysis Agric. Biotechnol. 10, 156–160. doi: 10.1016/j.bcab.2017.03.002

[B25] Lalević D. Ilić Z. S. Stanojević L. Milenković L. Šunić L. Kovač R.. (2023). Shade-induced effects on essential oil yield, chemical profiling, and biological activity in some Lamiaceae plants cultivated in Serbia. Sci. Hortic. 9, 84. doi: 10.3390/horticulturae9010084

[B26] Li G. Y. Song X. W. Sheng W. W. Wang M. (2017). Discussion on medicinal history and germplasm resource of *Clinopodium Herba* . Zhonghua Yi Shi Za Zhi 47, 149–151. doi: 10.3760/cma.j.issn.0255-7053.2017.03.003 28810344

[B27] Liu H. Wang X. Zhang B. Han Z. Wang W. Chi Q.. (2020). Concentration and distribution of selenium in soils of mainland China, and implications for human health. J. Geochem Explor. 220, 106654. doi: 10.1016/j.gexplo.2020.106654

[B28] Mansinhos I. Gonçalves S. Romano A. (2024). How climate change-related abiotic factors affect the production of industrial valuable compounds in Lamiaceae plant species: a review. Front. Plant Sci. 15. doi: 10.3389/fpls.2024.1370810 PMC11266143 39049861

[B29] Mattana E. Picciau R. Puddu S. Cuena Lombraña A. Bacchetta G. (2016). Effect of temperature and cold stratification on seed germination of the Mediterranean wild aromatic *Clinopodium sandalioticum* (Lamiaceae). Plant Biosyst. 150, 846–850. doi: 10.1080/11263504.2016.1196760

[B30] Miao L. L. Zhou Q. M. Peng C. Meng C. W. Wang X. Y. Xiong L. (2019). Discrimination of the geographical origin of the lateral roots of *Aconitum Carmichaelii* using the fingerprint, multicomponent quantification, and chemometric methods. Molecules 24, 4124. doi: 10.3390/molecules24224124 31739601 PMC6891363

[B31] Ni M. Vellend M. (2023). Soil properties constrain predicted poleward migration of plants under climate change. New Phytol. 241, 131–141. doi: 10.1111/nph.19164 37525059

[B32] Nuñez T. A. Prugh L. R. Lambers J. H. R. (2023). Animal-mediated plant niche tracking in a changing climate. Trends Ecol. Evol. 38, 654–665. doi: 10.1016/j.tree.2023.02.005 36932024

[B33] O’Neill B. C. Kriegler E. Ebi K. L. Kemp-Benedict E. Riahi K. Rothman D. S.. (2017). The roads ahead: Narratives for shared socioeconomic pathways describing world futures in the 21st century. Global Environ. Change 42, 169–180. doi: 10.1016/j.gloenvcha.2015.01.004

[B34] Oliveira A. Lopes A. Niza S. (2020). Local climate zones classification method from Copernicus land monitoring service datasets: An ArcGIS-based toolbox. MethodsX 7, 101150. doi: 10.1016/j.mex.2020.101150 33304834 PMC7718173

[B35] Parmesan C. Hanley M. E. (2015). Plants and climate change: complexities and surprises. Ann. Bot. 116, 849–864. doi: 10.1093/aob/mcv169 26555281 PMC4640131

[B36] Parmesan C. Yohe G. (2003). A globally coherent fingerprint of climate change impacts across natural systems. Nature 421, 37–42. doi: 10.1038/nature01286 12511946

[B37] Peng D. L. Song B. Yang Y. Niu Y. Sun H. (2016). Overlapping leaves covering flowers in the alpine species *Eriophyton wallichii* (Lamiaceae): Key driving factors and their potential impact on pollination. PLoS One 11, e0164177. doi: 10.1371/journal.pone.0164177 27716786 PMC5055289

[B38] Phillips S. J. Anderson R. P. Schapire R. E. (2006). Maximum entropy modeling of species geographic distributions. Ecol. Model. 190, 231–259. doi: 10.1016/j.ecolmodel.2005.03.026

[B39] Phillips S. J. Anderson B. P. Dudik M. Schapire R. Blair M. E. (2017). Opening the black box: an open-source release of Maxent. Ecography 40, 887–893. doi: 10.1111/ecog.03409

[B40] Piotrowska-Weryszko K. Weryszko-Chmielewska E. Sulborska-Różycka A. Konarska A. Kubik-Komar A. (2024). Global warming contributes to reduction in the intensity of Artemisia pollen seasons in Lublin, central-eastern Poland. Ann. Agric. Environ. Med. 31, 185–192. doi: 10.26444/aaem/184726 38940101

[B41] Pshenichkina Y. A. (2022). Biology of *Scutellaria baicalensis* Georgi (Lamiaceae) from different ecological and geographical places of growth during introduction. Contemp Probl Ecol. 15, 653–658. doi: 10.1134/S1995425522060129 36533088 PMC9748882

[B42] Rahmanian S. Pouyan S. Karami S. Pourghasemi H. R. (2022). “Chapter 17 -Predictive habitat suitability models for *Teucrium polium* L. using boosted regression trees,” in Computers in Earth and Environmental Sciences. Ed. Pourghasemi H. ,. R. (Elsevier, USA), 245–254. doi: 10.1016/B978-0-323-89861-4.00029-4

[B43] R Core Team (2024). R: A language and environment for statistical computing (Vienna, Austria: R foundation for statistical computing). Available at: https://www.R-project.org/.

[B44] Tian X. M. Lv H. Xiang G. F. Peng J. Li G. F. He Y.. (2023). Influence of geographic origin and tissue type on the medicinal chemical compounds of *Semiliquidambar cathayensis* . PeerJ 11, e15484–e15484. doi: 10.7717/peerj.15484 37304883 PMC10252815

[B45] Wan B. L. Qin W. Zhang H. Yang M. X. Liu S. J. (2022). Prediction of the suitable area of *Clinopodium chinense* based on maximum entropy model and geographic information system. Chin. J. Inf. Traditional Chin. Med. 29, 1–4. doi: 10.19879/j.cnki.1005-5304.202201247

[B46] Wang Y. Du K. Wang Q. Yang X. Meng D. (2024). A multidimensional strategy for characterization, distinction, and quality control of two *Clinopodium* medicinal plants. J. Ethnopharmacol 327, 118019. doi: 10.1016/j.jep.2024.118019 38467319

[B47] Wang R. Yang H. Wang M. Zhang Z. Huang T. Wen G.. (2020). Predictions of potential geographical distribution of *Diaphorina citri* (Kuwayama) in China under climate change scenarios. Sci. Rep. 10, 9202. doi: 10.1038/s41598-020-66274-5 32513980 PMC7280263

[B48] Wu Q. J. (1848). The Illustrated Investigation of Plant Names and Facts. Zhejiang: Zhejiang People's Fine Arts Publishing House.

[B49] Wu Z. Y. Li X. W. (1977). Volume 66 in Flora of China (Beijing: Science press), 223–224.

[B50] Xu Y. D. Zhu R. F. Gao L. F. Huang D. J. Fan Y. Liu C.. (2023). Predicting the current and future distributions of *Pennisetum alopecuroides* (L.) in China under climate change based on the MaxEnt model. PLoS One 18, e0281254. doi: 10.1371/journal.pone.0281254 37014870 PMC10072476

[B51] Yang Y. Z. Ma L. Li Y. T. (2014). Research on the cultivation technology of "duan xue liu. Anhui Agric. Sci. 42, 11662–11663. doi: 10.13989/j.cnki.0517-6611.2014.33.022

[B52] Ye H. G. Li C. Y. Ye W. C. Zeng F. Y. Liu F. F. Liu Y. Y.. (2022). “Medicinal angiosperms of Labiatae,” in Common Chinese Materia Medica. Eds. Ye H. G. Li C. Y. Ye W. C. Zeng F. Y. (Springer, Singapore), 463–533. doi: 10.1007/978-981-16-5904-1_8

[B53] Yoon D. Shin W. C. Oh S. M. Choi B. R. Lee D. Y. (2022). Integration of multiplatform metabolomics and multivariate analysis for geographical origin discrimination of *Panax ginseng* . Food Res. Int. 159, 111610–111610. doi: 10.1016/j.foodres.2022.111610 35940805

[B54] Zhou Y. B. Newman C. Chen J. Xie Z. Q. Macdonald D. W. (2013). Anomalous, extreme weather disrupts obligate seed dispersal mutualism: snow in a subtropical forest ecosystem. Glob Chang Biol. 19, 2867–2877. doi: 10.1111/gcb.12245 23640765

[B55] Zou H. Chen B. R. Zhang B. Y. Zhou X. Y. Zhang X. Y. Zhang X. X.. (2023). Conservation planning for the endemic and endangered medicinal plants under the climate change and human disturbance: a case study of *Gentiana manshurica* in China. Front. Plant Sci. 14. doi: 10.3389/fpls.2023.1184556 PMC10410459 37564387

[B56] Zou H. Zhang B. Y. Chen B. R. Duan D. T. Zhou X. Y. Chen J. X.. (2024). A multi-dimensional "climate-land-quality" approach to conservation planning for medicinal plants: Take *Gentiana scabra* Bunge in China as an example. Ind. Crop Prod 211, 118222. doi: 10.1016/j.indcrop.2024.118222

